# The role of BMP4 in adipose-derived stem cell differentiation: A minireview

**DOI:** 10.3389/fcell.2022.1045103

**Published:** 2022-10-21

**Authors:** Abdul Malik Setiawan, Taty Anna Kamarudin, Norzana Abd Ghafar

**Affiliations:** ^1^ Department of Anatomy, Faculty of Medicine, Universiti Kebangsaan Malaysia, Kuala Lumpur, Malaysia; ^2^ Department of Anatomy, Maulana Malik Ibrahim State Islamic University, Malang, Indonesia

**Keywords:** BMP4, ADSC, lineage, differentiation, signaling

## Abstract

Bone morphogenetic protein 4 (BMP4) is a member of the transforming growth factor beta (TGF-β) superfamily of cytokines responsible for stem cells’ commitment to differentiation, proliferation, and maturation. To date, various studies have utilized BMP4 as a chemical inducer for *in vitro* differentiation of human mesenchymal stem cells (MSCs) based on its potential. BMP4 drives *in vitro* differentiation of ADSC *via* TGF-β signaling pathway by interactions with BMP receptors leading to the activation of smad-dependent and smad-independent pathways. The BMP4 signaling pathways are regulated by intracellular and extracellular BMP4 antagonists. Extracellular BMP4 antagonist prevents interaction between BMP4 ligand to its receptors, while intracellular BMP4 antagonist shutdowns the smad-dependent pathways through multiple mechanisms. BMP4 proved as one of the popular differentiation factors to induce ADSC differentiation into cell from mesodermal origin. However, addition of all-trans retinoic acid is also needed in trans-differentiation of ADSC into ectodermal lineage cells. Suggesting that both BMP4 and RA signaling pathways may be necessary to be activated for *in vitro* trans-differentiation of ADSC.

## 1 Introduction

Adipose-derived stem cell (ADSC) is a type of mesenchymal stem cell (MSC) found in the vascular stroma of human fat tissue (Gojanovich et al., 2018). MSCs have the potential for self-renewal and differentiation into various cell types, including osteoblasts, chondrocytes, myoblasts, adipocytes, and tenocytes. MSCs are found in human adipose tissue, bone marrow, umbilical cord and amniotic fluid (Sarugaser et al., 2009). It has the ability to differentiate into osteoblast, adipocytes, myocytes, neural cells, chondroblast, skin and corneal cells *in vitro* (Dominici et al., 2006). The specific cytokines responsible for initiating and stimulating MSCs differentiation are members of the TGF-β superfamily, including Activins, Nodals, bone morphogenetic proteins (BMPs), and growth and differentiation factors (GDFs) (Weiss and Attisano, 2013).

ADSC has self-renewal and multipotent properties that make it a popular candidate for stem cell treatment (Argentati et al., 2018). Many studies have succeeded to differentiate ADSC into various cells of mesodermal, ectodermal and endodermal origin (Amer et al., 2018; Yogi et al., 2021; Zhou et al., 2022). *In vitro* differentiation of MSC generally performed using certain combination of differentiation factors that belong to TGF-β superfamily such as BMP4 and BMP7 (Grafe et al., 2018; Liang et al., 2022). There are more than 30 types of BMP that are the members of the TGF-β superfamily, but BMP4 is the most prolific member due to its dominant function in many cellular activities during human development and in life. Although, most studies focused on the effects of BMP4 on MSC differentiation, only limited number of studies have specifically discussed the role of BMP4 in ADSC differentiation.

BMP4 is a member of the TGF-β superfamily of cytokines responsible for stem cells’ commitment to differentiation, proliferation, and maturation (Katagiri and Watabe, 2016). Exogenous BMP4 have also been used as a chemical inducer to differentiate embryonic stem cell, mesenchymal stem cells and pluripotent stem cells into different type of cells *in vitro* (Hamid et al., 2018; Kamarudin et al., 2018; Lee et al., 2021). Different concentrations of exogenous BMP4 were used in ADSC differentiation ranging from 2.5 ng/ml to 25 ng/ml (Khazaei et al., 2022). However, little is understood about the BMP4’s signaling pathway and how it drives *in vitro* ADSC differentiation toward certain cell lineages. Thus, this review aims to explain and summarize the role of BMP4 signaling as well as its regulatory function in ADSC differentiation.

## 2 ADSC differentiation

ADSC is one of the most abundant MSC and it is relatively easy to procure compared to other sources of MSCs (Gojanovich et al., 2018). It possesses self-renewal, multipotent properties and regenerative capacity in tissue repair that make it a popular source of stem cells among researchers (Mazini et al., 2019). Potentially, 1 g of adipose tissue contains approximately 0.5 × 10^4^—2 × 10^5^ stem cells, whereas only 60—600 MSCs collected from 1 ml of bone marrow aspirates (Li et al., 2011; Baer and Geiger, 2012). Clinical applications of ADSC in stem cell therapy has been studied in the last decades. *In vivo* studies have successfully differentiated ADSC into neuronal cells in animal model, as well as chondrocyte and epithelial cells in human study (Meng et al., 2021; Wu et al., 2021). However, *in vitro* differentiation of ADSC becomes the main focus in stem cell studies since it allows the researcher to expand the ADSC population, survival rate and differentiation capacity toward multiple lineages.

*In vitro* differentiation of ADSC can be achieved through several methods such as co-culturing the stem cells with the signals-producing cells or by chemical induction *via* differentiation medium supplementation (Theerakittayakorn et al., 2020). Supplementation of differentiation factors has been shown to increase the expression of key transcription factors and promote ADSC differentiation (Almalki and Agrawal, 2016). The transcription factors responsible for ADSC differentiation are CCAAT/enhancer-binding proteins (C/EBPs) β and δ (encoded by CEBPB and CEBPD, respectively). The expression of runt-related transcription factor 2 (Runx2) is considered to be the driver of osteogenic commitment (Weiss and Attisano, 2013), while during chondrogenic differentiation, the expression of the critical chondrogenic transcription factor SRY-box 9 protein (Sox9) is required. These suggest, overexpression of certain transcription factors may promote ADSC differentiation into certain lineage (Almalki and Agrawal, 2016).

ADSC has the potencial to differentiate into cells types of mesodermal, ectodermal and endodermal origin. *In vitro* differentiation of ADSC into three mesodermal lineages of osteoblasts, adipocytes and chondroblasts can be performed in standard culture condition (Dominici et al., 2006). However, certain chemical inducers are needed ADSC into other mesodermal lineages such as myocytes and endothelial cells. BMP4 was used in combination with other growth factors such as TGF-β1, rhVEGF and bFGF to differentiate human ADSC into cardiomyocytes and smooth muscle cells (Shang et al., 2019; Hasani et al., 2020; Yogi et al., 2021). This suggests that BMP4 and other members of TGF-β superfamily initiate ADSC cell commitment and differentiation into mesodermal lineage *via* TGF-β and BMP signaling pathways (Mullen and Wrana, 2017).

BMP4 have been reported to successfully trans-differentiate human ADSC into keratinocytes (Khazaei et al., 2022). However, in order to induce trans-differentiation, BMP4 was used in combination with retinoic acid (Maeda et al., 2017; Petry et al., 2018). Thus, implying that TGF-β and BMP signaling pathways are not the only pathways that initiate cell commitment and differentiation of ADSC into cells from ectodermal origin. Previous study showed that retinoic acid suppressed the activity of Oct4 and Nanog, a pluripotency gene, and increase the expression of Nestin, an ectodermal marker gene (Zhang et al., 2015). *In vitro* differentiation of ADSC into ectodermal lineage were also possible by co-culturing ADSC with supporting cells (Nieto-Miguel et al., 2013). However, little is understood on which signaling pathway was involved in the co-culture method.

## 3 BMP4 structure and function

BMP4 is an endogenous protein molecule with double poly-peptide chains that are attached to one another by a single disulfide bond to form a covalent bond (Miljkovic et al., 2008). BMP4 is responsible for ADSC commitment during differentiation into more specialized cells and caused the committed stem cells to proliferate (Grafe et al., 2018). In addition, BMP4 exposure to human induced pluripotent stem cell (hiPSC) in early phase was responsible for ectodermal differentiation into corneal surface cells (Kobayashi et al., 2020). *In vitro* studies showed an increase of endogenous BMP4 expression in the early stage of differentiation. However, at the end of the differentiation phase, the expression of BMP4 was gradually decreased (Wong et al., 2019).

In the last decade, researchers have been investigating the potential of exogenous BMP4 as a chemical inducer for stem cells differentiation to induce *in-vitro* differentiation of MSCs (Kang et al., 2009; Weiss and Attisano, 2013). Exogenous BMP4 is a recombinant human BMP4 protein that is used as a chemical inducer for *in vitro* stem cell differentiation. Several studies proved that exogenous BMP4 could drive MSC differentiation into mesodermal, endodermal, and ectodermal lineages *in vitro*. Yogi et al. (2021) showed that BMP4 could be used to differentiate ADSC into vascular smooth muscle cells, while Kamarudin et al. showed that BMP4 could drive embryonic and induced-pluripotent stem cells differentiation into corneal epithelial-like cells, which belong to ectodermal lineage (Kamarudin et al., 2018).

Studies in animal models showed that *in vitro* differentiation capacity of exogenous BMP4 in mouse embryonic stem cells (ESC) was dose-dependent. Nakayama et al. (2003) showed in their study that 50 ng/ml of BMP4 was able to promote chondrogenesis of mouse ESC at a higher rate compared to 20 ng/ml, while the 5 ng/ml dose was not effective. In addition, Taha et al. (2006) reported that 100 ng/ml of BMP4 was able to induce adipogenesis at a higher rate in mouse ESC compared to 50 and 10 ng/ml doses. Based on the multilineage differentiation capacity of BMP4 as a chemical inducer, BMP4 could potentially play as a master controller of stem cell differentiation.

## 4 BMP4 signaling pathways and regulatory mechanisms in stem cell differentiation

**FIGURE 1:**
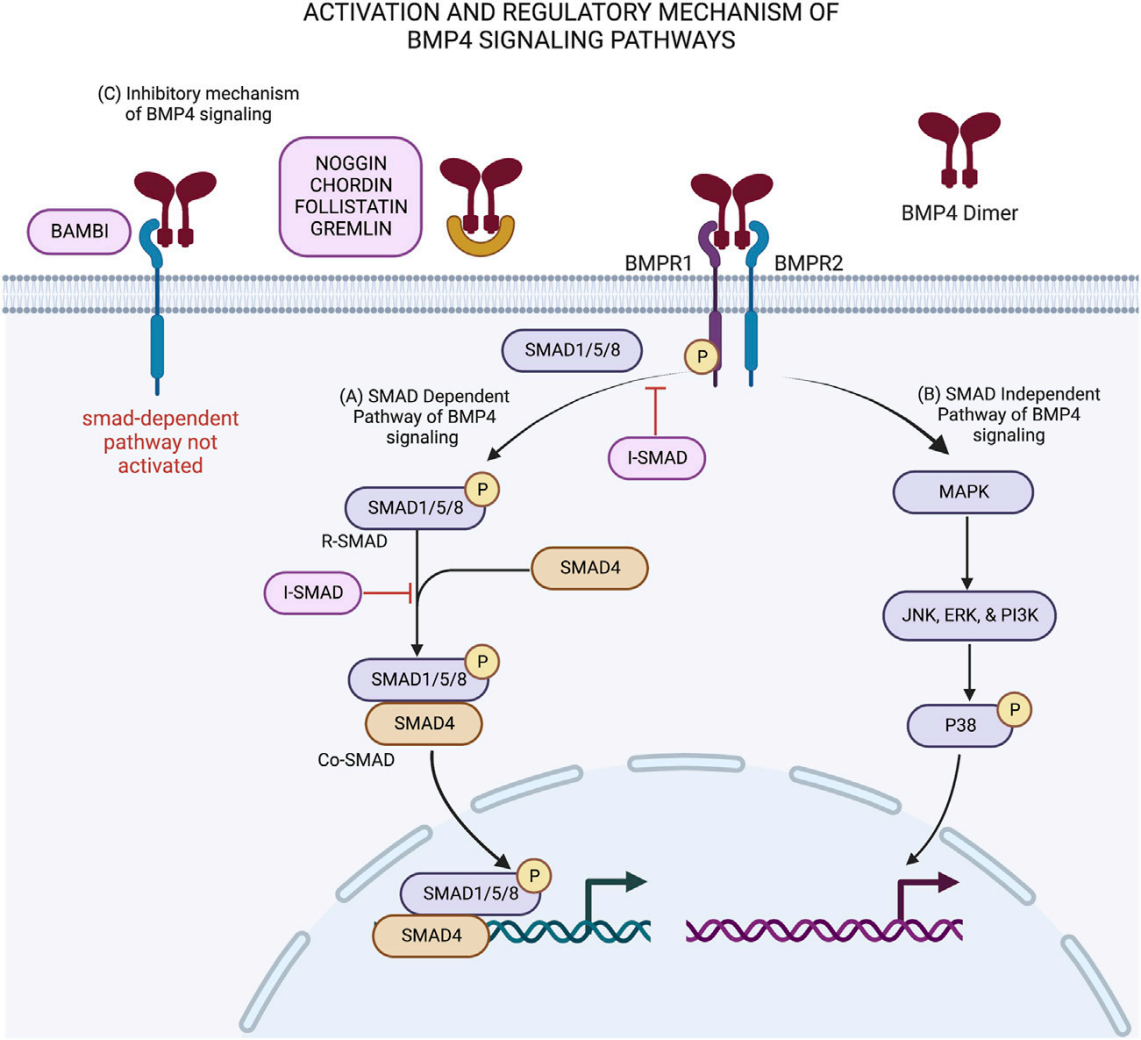
BMP4 signaling pathways and its’ regulatory mechanisms in stem cell differentiation: **(A)** Activation of smad-dependent pathway is initiated when BMP4 dimer binds to BMPR1/2 complex. This interaction activates the phosphorylation of receptor-associated SMADs (SMAD 1/5/8) by BMPR1 to produce R-smads. R-smads then recruit SMAD4 to form Co-smad and translocate into the nucleus leading to downstream signaling. **(B)** In smad-independent pathway, BMPR1 activates MAP kinase and acts as transcription factors inside the nucleus. Both pathways induce cell commitment and differentiation. **(C)** BMP4 signaling pathways are regulated by intracellular and extracellular antagonist molecules. Extracellular BMP antagonist (noggin, chordin, follistatin, gremlin and BAMBI) binds to BMP4 dimer ligand and prevent the interaction of BMP4 ligand with its BMP receptors leading to no activation of smad-dependent pathway. On the other hand, intracellular BMP antagonist, known as inhibitory smad (I-smad), shutdown the activated smad-dependent pathway by preventing the phosphorylation of r-smads and block the interaction between R-smads with Co-smads. This schematic was created with BioRender.com.

BMP4 activation and inhibition is regulated by its upstream signal pathways (Shen et al., 2020). Wnt/β-catenin signal pathways play a critical role in the expression BMP4 proteins. The increase of β-catenin expression leads to the increase of BMP4 expression (Shu et al., 2005). Cell commitment and proliferation of human progenitor cells is initiated through the activation BMP signaling cascade that leads to the expression of transcription factors in the nucleus that are needed for cell proliferation and differentiation (Cole et al., 2016). As presented in Figure 1, the commitment for cell differentiation of ADSC is activated when glycosylated BMP4 dimers bind to a type I (BMPRI) and type II (BMPRII) BMP receptors complex located on the cell surface. Following the ligand-receptor complex formation, activated BMPRII phosphorylates and activates BMPRI. The BMPRI then starts to phosphorylate the receptor-regulated Smads (R-SMAD). The R-SMAD consist of (SMAD1, SMAD5, and SMAD8). In addition, BMP4 also initiates signaling *via* a non-canonical smads-dependent pathway. The capacity of BMP4 to bind to multiple receptors is strongly influenced by several antagonists like NOG, CHRD, GREM and FST (Dexheimer et al., 2016).

Figure 1 shows two types of signaling cascades involved in the activation BMP4 signaling pathways. In the smad-dependent pathway (Figure 1A), phosphorylated SMAD1/5/8 known as R-SMAD then recruit SMAD4 and translocate it from cytoplasm to cell nucleus. Inside the nucleus of the ADSC, the heterodimer complex between R-SMAD and SMAD4 (Co-SMAD) with other transcription factors control target gene expression to produce proteins that are needed for the differentiation process (Aashaq et al., 2022). On the other hand, smad-independent pathway or non-canonical smad-dependent pathway (Figure 1B) is initiated as BMP receptor complex activates mitogen activated protein kinase (MAPK) and acts as transcription factors inside the nucleus through their downstream molecules with similar function as Co-SMAD in the smad-dependent pathway (Miyake et al., 2020).

The regulatory mechanism of BMP4 is facilitated by extracellular and intracellular BMP4 antagonists as presented in Figure 1C. BMP4 antagonists regulate the BMP4 signaling activity by providing negative feedback loop in order to decrease cell commitment and differentiation (Cole et al., 2016). Several extracellular BMP4 inhibitors, such as NOGGIN, CHORDIN, FOLISTATIN, and GREMLIN bind directly to BMP4 heterodimer and prevent proper interaction between BMP4 ligand and its receptor (Glister et al., 2019; Phan-Everson et al., 2021). A BMP4 extracellular inhibitor, called BAMBI, acts as a pseudo receptor on the cell surface. BAMBI is a transmembrane protein that shares similar structure with BMPRI. The binding of BMP4 dimers with BAMBI prevents proper interaction between BMP4 ligand and BMP receptor, hence halting the downstream BMP4 signaling activity (Pfeifer et al., 2016).

Intracellular BMP4 inhibitors includes inhibitory smads (SMAD 6 and 7), also known as I-SMAD and GREMLIN interfere in smad-dependent pathway *via* multiple mechanisms. The structure of I-SMAD protein consists of N domain and MH2 domain, whereas the MH2 domain is conserved in R-SMAD and Co-SMAD as well (Miyazawa and Miyazono, 2017). I-SMAD prevents the attachment of SMAD 1/5/8 with BMPRI and prevents the R-SMAD phosphorylation. I-SMAD also prevents R-SMAD to recruit SMAD4. GREMLIN acts as BMP4 extracellular antagonist when secreted by other cells and acts as intracellular BMP4 antagonist when co-expressed in the same cell with BMP4 precursor. GREMLIN binds to BMP4 precursor and prevents the secretion of mature BMP4 outside the cell (Li and Wu, 2020).

## 5 Induction of ADSC differentiation by BMP4

### 5.1 Mesodermal differentiation

Mesoderm is the middle layer of the three germinal layers, and makes up a large portion of the human body, including mesenchymal tissues such as bone, cartilage, adipose, fibroblasts, myocytes and endothelial (Evseenko et al., 2010). As a progenitor cell that reside in mesodermal origin of adipose tissue, ADSC would spontaneously differentiate and proliferate into adipocytes (Yang et al., 2018). However, ADSC has the capacity to differentiate into other mesodermal origin cell such as chondrocytes and osteoblasts in laboratory condition without the use chemical inducers such as BMP4 (Dominici et al., 2006). In contrast, *in vitro* differentiation of ADSC into other mesodermal origin cells such as myocytes and endothelial cells cannot be performed without the help of BMP4 as a chemical inducer. As presented in Table 1, several studies used BMP4 as an induction factor to differentiate ADSC into vascular endothelial cells and cardiomyocytes.

**TABLE 1:** Differentiation of ADSC into cells from mesodermal and ectodermal origin.

Cell type	Differentiation medium	Time	Cell markers	References
Mesodermal lineage				
Vascular smooth muscle cells	DMEM +1% FBS, 5 ng/ml TGF-β1, 2.5 ng/ml BMP4	7 days	The expression of α-SMA, Calponin, SM22α, MHC	(Wang et al., 2010)
Vascular smooth muscle cells	LG-DMEM + 1% FBS, 5 ng/ml TGF-β1, and 2.5 ng/ml BMP4	7 days	The expression of α-SMA, Calponin, SM22α, MHC	(Aji et al., 2016)
Vascular endothelial cell	EGM-2 + 2% FBS, 50 ng/ml rhVEGF, 100 ng/ml BMP4, under hypoxia (2% O2)		CD31, VEGF-R2, and VE-cadherin	(Shang et al., 2019)
Cardiomyocytes	DMEM +10% FBS, 10 ng/ml bFGF, 20 ng/ml BMP4	4 days	The expression of GATA4, MEF2C, MLC2A, MLC2V, a-Actinin, Troponin I	(Hasani et al., 2020)
Vascular smooth muscle cell	DMEM +1% FBS, 5 ng/ml TGF-β 2.5 ng/ml BMP4	7 days	The expression of α-SMA, SM22α, calponin and SM-MHC	(Lin et al., 2020)
Vascular smooth muscle cells	DMEM +1% FBS, 5 ng/ml TGF-β 2.5 ng/ml BMP4	4 days	The expression of -SMA, Caldesmon, SM22α, MHC	Yogi et al. (2021)
Ectodermal lineage				
Keratinocyte	DMEM +0.5 nM (17 ng/ml) BMP-4, 0.3 mM ascorbic acid 2-phosphate, 0.5 ug/ml hydrocortisone, 5 nM (1.5 ng/ml) retinoic acid, 20 ng/ml EGF	7 days	The expression of pan-cytokeratin, CK1, CK14, CK18, Involucrin	(Petry et al., 2018)
Keratinocytes	DMEM +1 μM All-trans retinoic acid, 25 ng/ml BMP4, co-culture with human dermal fibroblasts (NHDFs) (5 × 104 cells/ml) keratinocyte serum-free medium (KSFM) + co-culture with NHDFs	4 days	Cytokeratin 10 (K10), Type VII collagen (Col7)	(Maeda et al., 2017)
		7 days		

A study by Wang et al., in 2010 reported that ADSC could be differentiated to smooth muscle cells by using differentiation media containing 1% FBS, 5 ng/ml TGF-β1, 2.5 ng/ml BMP4. Aji et al., Lin et al., and Yogi et al. used the same differentiation media and reported similar outcomes with upregulation of α-SMA, calponin, SM22α, and MHC expressions. In addition, Aji et al. (2016) suggested that following the BMP4 signaling pathway activation, the multifunctional Ca^2+^/calmodulin-dependent protein kinase II (CaMKII) plays a significant role in smooth muscle differentiation of ADSC as a downstream effect of the induction. They also demonstrated that the Krüppel-like factor 4 (KLF4) protein regulated the smooth muscle differentiation that was induced by BMP4 signaling (Aji et al., 2017). Furthermore, Lin et al. (2020) revealed that N6-adenosine methyltransferases (Mettl3), an N6-methyltransferase that methylates adenosine residues at the N6 position of some RNAs, was upregulated following BMP4 induction. The expression of Mettl3 was similar to CaMKII, in which suppression of this gene expression will in turn decrease the expression of vascular smooth muscle markers.

Vascular endothelial cells and cardiomyocytes are mesodermal origin cells that have successfully undergone *in vitro* differentiation from ADSC using BMP4. In 2019, Shang et al. (2019) showed that a combination of 100 ng/ml BMP4 and 50 ng/ml vascular endothelial growth factors (VEGF) could induce ADSC differentiation into vascular endothelial cells under hypoxic conditions. In another study by Hasani et al. (2020), ADSC had successfully differentiated into cardiomyocytes using only 20 ng/ml BMP4 and 10 ng/ml basic fibroblast growth factor (bFGF) as chemical inducers. Although 20 ng/ml BMP4 alone could induce cardiomyocytes differentiation, the combination of BMP4 and bFGF was reported to have improved the expression of cardiac-specific markers. This suggests that members of FGF family could induce cardiomyocytes differentiation through paracrine and endocrine FGF signaling pathways (Itoh et al., 2016). Upon FGF ligand and signaling tyrosine kinase FGF receptors (FGFRs) binding, four transduction factors, RAS-MAPK, PI3K-AKT, PLCγ, and STAT, are activated and promote differentiation, proliferation and survival of cardiomyocytes (Ornitz and Itoh, 2015).

### 5.2 Ectodermal differentiation

Ectoderm is the outermost and the most superficial layers in human body that includes epithelial and ocular surfaces (Shahbazi, 2020). BMP4 was used as differentiation factor in several studies to differentiate ADSC into an ectodermal origin cell, keratinocytes. As summarized in Table 1, Maeda et al. (2017) and Petry et al. (2018) used combination of BMP4 (17 & 25 ng/ml) and 1.5 ng/ml retinoic acid (RA) to induce keratinocytes differentiation. However, Maeda et al. (2017) incorporated human dermal fibroblasts in a co-culture environment as a comparison to chemical induction method. Both studies had successfully showed the increase of cytokeratin expression (CK1, CK8, CK10 and CK18) in both systems (Maeda et al., 2017; Petry et al., 2018). In addition, Maeda et al. showed that the expression of CK10 and Col7 was significantly upregulated in the BMP4 and RA supplemented medium compared to the fibroblast co-culture system which was not supplemented with BMP4 and RA (Petry et al., 2018). Based on those findings, ectodermal differentiation of ADSC requires higher concentration of BMP4 compared to mesodermal differentiation. In Addition, ectodermal differentiation might not be activated only *via* the TGFβ signaling pathway but also through retinoic acid (RA) signaling pathways. This is in agreement with the reports that suggested RA signaling pathway led to the Wnt signaling inhibition and promoted the non-neural ectodermal differentiation and proliferation of the corneal epithelial cells (Metallo et al., 2008; Ghyselinck and Duester, 2019; Kobayashi et al., 2020). Hence, our review suggests that BMP4 is vital for ectodermal differentiation in ADSC and it is closely supported by RA.

## 6 Conclusion

ADSC differentiation is a process that involves various signaling pathways. During this process, different pathways play a major role depending on the target cell lineage. Mesodermal differentiation of ADSC is more efficient compared to ectodermal trans-differentiation due to the origin of ADSC from the mesodermal germ layer. Mesodermal differentiation requires lesser dose of BMP4 compared to ectodermal trans-differentiation. However, there is still lack of standardized optimal BMP4 concentration needed in order to induce ADSC differentiation into certain lineage. Therefore, future studies to elucidate the optimal concentration of BMP4 as a differentiation factor is crucial. Unfortunately, no report was found on BMP4 usage in endodermal differentiation of ADSC to date. Furthermore, no *in vivo* study reports on the effect of BMP4 in ADSC differentiation. Thus, more studies are still needed to explain the role of BMP4 in ADSC differentiation which is still at an early stage before it could be applied clinically.

BMP4/TGF-β signaling pathway plays a significant role in both mesodermal and ectodermal differentiation of ADSC. BMP4 alongside RA and tissue-specific growth factors were reported as potent differentiation factors in ADSC differentiation either in mesodermal differentiation or ectodermal trans-differentiation. Thus, a detailed and in-depth review and research work is important to explain the interplay amongst the different pathways involved especially in ADSC trans-differentiation in the future.
